# Expectations Versus Reality: Long-Term Research on the Dog–Owner Relationship

**DOI:** 10.3390/ani10050772

**Published:** 2020-04-29

**Authors:** Esther M. C. Bouma, Lonneke M. Vink, Arie Dijkstra

**Affiliations:** Department of Social Psychology, Faculty of Behavioural and Social Sciences, University of Groningen, 9712 CP Groningen, The Netherlands; l.m.vink@rug.nl (L.M.V.); arie.dijkstra@rug.nl (A.D.)

**Keywords:** self-efficacy, dog ownership history, relationship, longitudinal, satisfaction, canine problematic behavior

## Abstract

**Simple Summary:**

People who plan to own a dog have expectations about what this experience will be like and are initially in the motivational phase of dog-ownership. Their expectations about taking care of a dog and the benefits of this relationship determine if they will acquire a dog. Once they have acquired a dog, their expectations are tested against reality; they are then in the experience phase of dog-ownership. In this phase, the owner’s relationship with their dog develops, which may be pleasant and satisfying. However, when problems appear, the owner may experience dissatisfaction with their dog. In this study, 183 people who were planning to acquire a dog answered questions before and after acquisition of their dog. How their expectations and beliefs changed over time depended on whether the participants had experience with dogs (owning a dog presently, in the past, or never). In the first six months of ownership, especially for people with no prior experience with dogs, the owners had to adapt their expectations and beliefs. In the subsequent year, only a few differences based on dog ownership history were found. To conclude, the perceptions of dog ownership do change over time, but after testing such perceptions with reality, the perceptions become stable after six months.

**Abstract:**

In the framework of the early prevention of problems in the owner–dog relationship, it is important to have a broad perspective on the development of this relationship over time, starting before people actually acquire a dog. People who currently (or previously) own(ed) a dog can rely on their experiences when considering a new dog, while this knowledge is unavailable to first time dog-owners. In this study, we explore how self-efficacy, social comparison, perceptions about the (dis)advantages of ownership and commitment to the dog (so-called social cognitive factors), problematic canine behaviors, perceived costs, and satisfaction with the dog change over time. We examine changes from the motivational phase of relationship development (before acquisition of the dog) into the experience phase (six and twelve months after acquisition of the dog). We explore if patterns are different in experienced (previous (*n* = 73) and current (*n* = 80)) versus unexperienced (first time (*n* = 30) dog owners. The respondents filled in three online questionnaires—once before and twice after acquisition of their dog. From T0 (before acquisition of the dog) to T1 (having the dog for six months) participants (especially those with no ownership experience) had to adjust their perceptions about dogs and dog ownership. Experiencing the relationship for an additional year (from T1 to T2) barely changed the social cognitive factors, satisfaction, and perceived costs. A small decline in problematic canine behaviors was present among the experienced dog owners between T1 and T2. To conclude, perceptions about dogs and dog ownership change over time, but after testing these perceptions with reality, they become stable after about six months.

## 1. Introduction 

Since companionship is the most common reason for owning a dog in the Western world [1,2], the quality of the owner–dog relationship is of great importance as this relationship will influence the owner’s satisfaction with their dog. Dog ownership is associated with increased physical activity, reduced stress levels, companionship, social support, and increased social interactions with people [3], although not all beneficial effects have been replicated. Although less well described, there are also negative consequences of dog ownership, such as the detrimental effect of a pet’s death on owner wellbeing [4], the development of allergies and asthma [5,6], and an increased risk of dog bites, especially among children [7]. 

The consequences of an unrealistic perspective on dog ownership benefits can affect the human–dog relationship in a negative manner. Underestimation of the necessary investments, such as daily walking, increased responsibility, and obedience training [8], can result in behavioral issues for the dog (e.g., difficulties in training, soiling, and aggressive behavior [9,10]). Unwanted (problematic) behaviors will negatively influence this relationship [1,11] and are one of the most commonly mentioned reasons for the relinquishment of dogs to shelters (together with insufficient veterinary services, unmet expectations, and a lack of participation in obedience classes [12]). When expectations are skewed towards the positive aspects of dog-ownership and investments (both financially and time-wise) are underestimated, a mismatch between expectations and reality is likely to occur. As previously shown, the likelihood of an owner’s satisfaction with a dog increases when the needs and expectations of the owner are compatible with the behavior of the dog [13,14].

Expectations are influenced by experience and knowledge about dog behaviors and needs. One would assume that previous dog owners can rely on their previous experiences when considering the acquisition of a new dog. Previous research [15] explored the association between prior animal experience and animal-care knowledge before adoption and showed that people with more knowledge about animal care, health, behavior, training, and costs had more realistic expectations about dog ownership than people with less knowledge. A large cross-sectional Australian study showed that previous or current dog owners had reduced odds of expecting challenges (such as responsibility and training) and greater odds of expecting benefits than first-time dog owners [8]. These results are contrary to those of O’Conner and colleagues [15], who showed that the ownership experience is related to a higher awareness of the efforts required to take care of a dog. Thus, previous dog ownership does not automatically result in realistic expectations; it is possible that people who experienced a relationship with a dog in the past will be overly optimistic, while first-time owners might be overly cautious.

Although the owner–dog relationship is influenced by both canine and human characteristics [16], research suggests that the owner plays a very important part in the affective state and subsequent behavior of the dog [17]. The behaviors of owners toward their dogs are influenced by multiple factors [18], such as the owner’s attitudes and beliefs about dogs, previous experiences with dogs, perceptions of society and peers, and biased views on personal knowledge and skills, as well as self-efficacy, which may change over time. Understanding people’s attitudes and behaviors towards animals is challenging and complex but of great importance if we seek to prevent problems. It is, therefore, important to study the development of the owner–dog relationship over time, starting before the acquisition of a dog. To understand the psychology of this relationship’s development, the owner’s perceptions (e.g., advantages and disadvantages) needs to be understood since these perceptions influence owners’ feelings and behaviors towards their dogs. Perceptions are based on experience, learning from others, and comparing oneself to others, and can be conceptualized as social cognitive determinants [19,20,21].

There are several different types of social cognitive determinants. Firstly, people have social cognitive perceptions related to the punishments and rewards contingent on owning a dog. In this study, we assess the perceived advantages that people expect and experience from their dog, such as companionship, social support, or feeling safe. In addition, we assess the perceived commitment to the dog, which represents the quality and, therefore, the gratification derived from the relationship. These reward-related social cognitive determinants result in the general tendency to “approach” the dog, to feel positive about the dog and the relationship, and to invest in the relationship. Besides expectations of rewards, we assess the perceived disadvantages that comprise the expected drawbacks that follow from this relationship, such as financial or time investments. These punishment-related social cognitive determinants yield the general tendency to “avoid” the dog, to feel negative about the dog and the relationship, and to minimize investments.

Secondly, people have social cognitive perceptions of their perceived ability to develop a satisfactory relationship with a dog, which will provide them with advantages and help them cope with the disadvantages. When people feel they are able to master the necessary skills for dog ownership, they will persevere and invest more into the relationship. People can estimate their ability to handle a dog and prevent problems based on their own experience (enactive learning), which we refer to as self-efficacy. This implies that people with little experience must use proof from other domains in their life, such as how well they can handle relationships in general, or by observing other people interact with a dog (vicarious learning). Another way of estimating one’s own ability is by making social comparisons [22], which are highly important in the process of self-evaluation [23]. In social comparisons, people compare their own ability with the abilities of other specific people (e.g., their neighbors) or in a more generalized manner (e.g., a constructed mental image of dog-owners in general). An overestimation of abilities can be functional when it support one’s confidence [24], which may lead to greater perseverance and investment, such as greater patience towards the dog’s behavior or consistency while training the dog.

**Figure 1:**
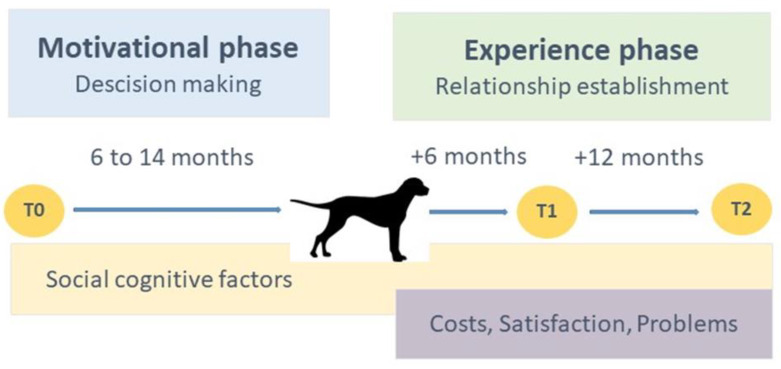
Two-phase model of the owner–dog relationship establishment and the research design of the present study.

Inspired by the Health Action Process Approach theory [25], we propose a two-phase model for the establishment of the owner–dog relationship (see Figure 1). In the first phase, the social cognitive determinants comprise expectations about the dog and the relationship with the dog and culminate in a decision to acquire the dog. In the second phase, this relationship is actually experienced, which may lead to an adjustment of the earlier social cognitive expectations. The present longitudinal design makes it possible to explore to how these social cognitive determinants change over time, from before acquiring the dog (motivational phase) to owning the dog for six months (from T0 to T1—the early experience phase) and from six months to another twelve months (from T1 to T2—the extended experience phase).

Experiencing an actual relation with a dog might change the owners’ social cognitive determinants. Experiencing reality can adjust one’s expectations from the motivational phase in two ways: Those who are (overly) cautious might be pleasantly surprised, and those who were (overly) optimistic might be disappointed. In addition, social cognitive factors can influence the behavior of the owner towards the dog. For example, when people have low self-efficacy, meaning that they are uncertain about how to train and take care of the dog, they may behave inconsistently towards the dog (e.g., not reinforcing commands). When they perceive the weak advantages and strong disadvantages of the dog, they may diverge in their communication, avoid the dog, and become less sensitive to the welfare needs of the dog.

Moreover, in the experience phase, owner perceptions may change due to evaluations of the relationship with their dog. Dog owners’ perceptions of their investments relative to their experienced benefits may lead to certain levels of (dis)satisfaction with the dog and to a sense of how difficult it is to own the dog. In addition, canine behavioral problems may manifest (or disappear) over time. These three factors (satisfaction, perceived costs, and canine behavioral problems), are, therefore, monitored over time. We expect that the transitions over time will be different between unexperienced and experienced dog owners, since the latter can rely on their previous or current experiences with dogs.

In a previous study [26] that was based on the same two-phase model of owner–dog relationship establishment (Figure 1), we explored prospective owner behavior in the motivational phase and examined how social cognitive determinants are involved in preparing and actually deciding to acquire a(nother) dog. We showed that the quality of the decision-making process (indicated by the social cognitive determinants) influences the desirable (satisfaction) and undesirable outcomes (perceived costs and problematic behaviors) in the experience phase. We showed that greater self-efficacy before the acquisition of a dog is significantly related to fewer canine behavioral problems and greater satisfaction with the dog both six and eighteen months later. In addition, expecting relatively more disadvantages was significantly related to higher perceived costs and less satisfaction with the dog after six months.

The aim of the present study is to explore how perceptions of dog ownership and (un)desirable consequences change over time, both for experienced and unexperienced owners. Data from three waves of longitudinal data (baseline (T0) and two follow-ups at six (T1) and eighteen (T2) months after acquiring the dog) were analyzed. Social cognitive measures (self-efficacy, perceived (dis)advantages, social comparison, optimism, and commitment) were measured three times (once in the motivational phase and twice in the experience phase), while problematic behaviors of the dog, satisfaction with the dog, and perceived costs of dog ownership were measured twice (in the experience phase).

## 2. Materials and Methods

### 2.1. Recruitment and Procedure

A call was published on several websites inviting people who were “planning to acquire a dog within one year” to complete an online questionnaire. Participants were informed that they would also be asked to complete another questionnaire six and eighteen months after acquiring a dog. The call was placed on the websites and Facebook pages of Dutch organizations that provide information about dogs and dog ownership. The researchers were assisted in contacting these organizations by the Dutch Royal Association for the Protection of Dogs (Koninklijke Hondenbescherming), an organization dedicated to the welfare of dogs in the Netherlands. One Belgian organization posted the appeal to its website. We approached 92 pet shops in the Netherlands with the request to place flyers containing the same call on their counter; 82 agreed to do so.

The questionnaire was created on the Qualtrics platform. A link distributed to participants redirected them to the online questionnaire, which contained a notice that, by continuing to the next page, they automatically gave their informed consent. The notice also stated that the results of the questionnaire would be processed anonymously. The questionnaire was administered in Dutch. The Institutional Review Board of the University of Groningen Faculty of Behavioral and Social Sciences reviewed and approved the research (ppo-014-265).

### 2.2. Three Measurement Waves

The present data include a baseline measurement and two follow-up measurements (six and eighteen months after acquiring a dog). Of the 1418 people who started the questionnaire at T0, 44.2% (*n* = 627) completed it. To time the second measurement (T1) at six months after acquisition of the dog, participants were contacted three times (between 6 and 14 months) after the baseline measurement to ask whether they had acquired a dog. Respondents who acquired a dog within 14 months after the first questionnaire (T0) were invited to participate in the questionnaires at T1 and T2. Of those completing the baseline questionnaire, 44.2% (*n* = 277) completed the T1 questionnaire, and 30.8% (*n* = 233) completed the T2 questionnaire. Ten people acquired a new dog between T1 and T2; eight discarded the dog they acquired after T0, and two lost their dog due to death. To facilitate the interpretation of our results, we report only on the participants who completed all three questionnaires for the same dog (*n* = 183).

### 2.3. Attrition Analysis

The dropout rates from T0 to T1 and from T1 to T2 were substantial. Of the 627 participants at T0, only 183 provided full data at both T1 and T2. The characteristics of the 183 participants were compared to those of the 444 participants who were not included in the present study. Both groups were compared by gender, level of education (intermediate/low versus high) and experience with dogs (previous/current versus never), age, perceived advantages and disadvantages, and self-efficacy, but no differences were present. Participants who dropped out before T2 were slightly (but significantly) younger (mean = 39.9 years, SD = 14.0 versus mean = 43.0 years, SD = 12.7) than the participants who were included in the analyses.

### 2.4. Variables Measured at T0, T1, and T2

*Self-efficacy* [19] was assessed with two items using 10-point scales to determine how certain people were that they were able to: 1) Raise/train a dog and 2) take care of a dog. The scales could be answered from “not certain at all” (1) to “very certain” (10). The average item score was used as the scale score (T0: r = 0.71, T1: r = 0.67, T2: r = 0.64); the higher the score, the more confident the respondent was about his or her abilities to handle the dog satisfactorily. The r can be considered as the reliability of the scale, where, as a general rule of thumb, a reliability of around 0.70 is acceptable and a reliability around 0.80 is good [27].

According to social comparison theory [22], people have an innate tendency to compare themselves to others. To determine how dog owners perceive themselves in comparison to others in their ability to take care of a dog (*social comparison score*), we presented our participants with the following six propositions: "Compared to other dog owners, to what extent is it likely that you... 1) understand problems with your dog?; 2) discard your dog?; 3) are able to control your dog?; 4) are able to train your dog?; and 5) are able to take good care of your dog?”; as well as 6) “compared to other dogs (of the same breed, age, and gender), to what extent is it likely that your dog will bite someone?”. Answers could be rated on a scale from -3 (far below the average) to 3 (far above the average), based on the format in [28]. Before analysis, the scales were recoded (−3 to 1, −2 to 2, −1 to 3, 0 to 4, +1 to 5, +2 to 6, and +3 to 7), and the scores of propositions one, two, and six were transposed. The mean item score was used as a social comparison scale score (T0: α = 0.80, T1: α = 0.70, T2: α = 0.74). The higher the score on this scale, the higher the discrepancy between the owners’ perceptions about their abilities compared to others.

*Advantages of dog ownership* were assessed with 25 items to determine the expected positive effects of owning a dog. This scale was based on the advantages of owning a dog as reported in the literature [2,29,30] and on 10 in-depth interviews and observations of how dog owners talked about their dog(s). Item examples are: “My dog will make sure that I have company” and “My dog will make sure that I get more physical exercise”. All 25 items are listed in Table S1 of the Supplemental Materials section. *Disadvantages of dog ownership* were assessed with ten items on the expected negative effects of owning a dog. These items were based on the previously mentioned interviews and personal observations. Item examples are: “Because of the dog, I will have to plan my life more” and “Because of the dog, I will have more expenses”. All 10 items are listed in Table S2 of the Supplemental Materials section. Items related to both advantages and disadvantages were rated on a five-point scale (totally disagree (1), disagree (2), neither disagree nor agree (3), agree (4), and totally agree (5)). The mean item scores for advantages (T0: α = 0.92, T1: α = 0.91, T2: α = 0.91) and for disadvantages (T0: α = 0.73, T1: α = 0.78, T2: α = 0.75) were used as the scale scores. The higher the score, the more (dis)advantages of dog ownership the participants perceived.

*Commitment*, a scale validated for measuring the commitment between humans [31], was adapted to investigate the human psychological processes related to commitment to dogs. This scale consisted of seven items: 1) “If your dog doesn’t live up to your expectations, can you imagine discarding the dog to get another?” 2) “How important is your dog to you?”, 3) “Do you intend to keep the dog forever?”, 4) “How likely is it that you will discard your dog in the future?”, 5) “To what extent are you attached to your dog?”, 6) “How motivated are you to always keep your dog?”, and 7) “How much will you do for your dog?”. These questions were answered on a five-point scale with different options for each question. Item-specific anchors for the examples ranged from “totally not attached/much/certainly not” (1) to “very attached/much/certainly” (5). Items one and four were recoded to ensure a similar valence of the items. The mean item score was used as the expected commitment scale score (α = 0.66) at T0. At T1 and T2, the phrasing was slightly different and changed from “to what extent do you think you will be attached to your dog?” to “to what extent are you attached to your dog”. The higher the score on this scale, the more the participants expected to be committed to the new dog. The mean item score at T1 was α = 0.67, and at T2, it was α = 0.80).

### 2.5. Variables Measured at T1 and T2

*Canine behavioral problems* were assessed at T1 and at T2 with sixteen items, such as “bad manners while eating”, “aggression toward other people”, “aggression toward other dogs”, “inappropriate house soiling”, and “inappropriate biting”. For a full list of these behaviors, see Table S3 in the Supplemental materials section. Items were inspired by the C-BARQ [32] and problematic behavior categories described in Blackwell’s five minute Veterinary Consult Clinical Companion: Canine and feline behavior [33]. Respondents were asked about the frequency of each behavior (1 = never, 2 = sometimes, 3 = regularly, 4 = often, and 5 = very often). The higher the score, the higher the occurrence of problematic behaviors.

*Perceived costs* were assessed at T1 and T2 with the “Perceived Costs” subscale of the Monash Dog Owner Relationship Scale (MDORS) [34]. This scale consists of nine items, which respondents can rate from “totally disagree” (1) to “totally agree” (5). Examples of these items are: “I often feel that taking care of my dog is a heavy duty”, “My dog costs too much money”, and “I often feel that having a dog is more effort than pleasure”. The average item score was used as the scale score (α = 0.86 at T1 and α = 0.87 at T2). The higher the score, the more costs the respondent perceived to be associated with dog ownership.

*Satisfaction with the dog* was assessed at T1 and T2 with four items [13], which the respondents could rate on a scale from “totally disagree” (1) to “totally agree” (7). The items are: 1) “In general, I am very satisfied with the experiences I have with my dog”, 2) “There are moments when I regret my decision to acquire this dog”, 3) “There are several things I would like to change about my dog”, and 4) “I am satisfied with my dog the way he/she is”. Items two and three were recoded to ensure the similar valence of the items. The average item score was used as the scale score with a Cronbach alpha’s of 0.49 at T1 and 0.73 at T2. The higher the scale score, the greater the satisfaction with the dog.

### 2.6. Statistical Analyses

The patterns of change among the six social cognitive factors and the three outcome measures were examined with repeated measures general linear modeling (GLM). A significant interaction between the factors (e.g., self-efficacy) and ownership groups indicates a different pattern between the groups. When the patterns did not differ (i.e., had a similar shape but different overall levels), the main effect of the group was examined to see if the groups differed on average levels (regardless of time). Paired t-tests were used to examine the contrasts (rise or fall between two time points) within each group. Group differences were examined for each time point with an analysis of variance (ANOVA). Since gender, age, and level of education might affect the outcome variables [35], these characteristics were included as covariates in all models. We used the software package IMB SPSS (version 25) to analyze our data. A *p*-value below 0.05 is considered statistically significant.

## 3. Results

### 3.1. Participant Characteristics

Participants who acquired a dog within fourteen months after the baseline questionnaire (T0) and were still in possession of the same dog at T2 were included in the study. The final sample consisted of 183 participants, most of whom were female (87%) and 59% of whom had a high education level. The average age was 43 years (with a standard deviation of 12 years); 21% were below 30 years; 36% were between 30 and 45, and an additional 36% were between 45 and 60 years (7% were over 60 years). Almost three-quarters (70%) did not work with dogs professionally. The respondents were mainly couples without (living at home) children (45%) or part of a family (35%); only 15% were single, and 6% shared a household with other adults. The majority (76%) obtained a puppy and not an adult dog. Regarding previous experience with dogs, 16% had never owned a dog before, while 44% had owned one (or more) dogs at T0. About 80% of the respondents attended a dog-training course (mostly obedience training and/or puppy courses).

### 3.2. Descriptive Elements

Table 1 shows the characteristics of the three dog ownership groups and, if present, the differences between the groups. First-time dog owners (indicated as “first” in Table 1) were younger compared to the owners who owned a dog in the past (“previous”) and those who already owned another dog at the start of the longitudinal study (“current”).

**Figure 2:**
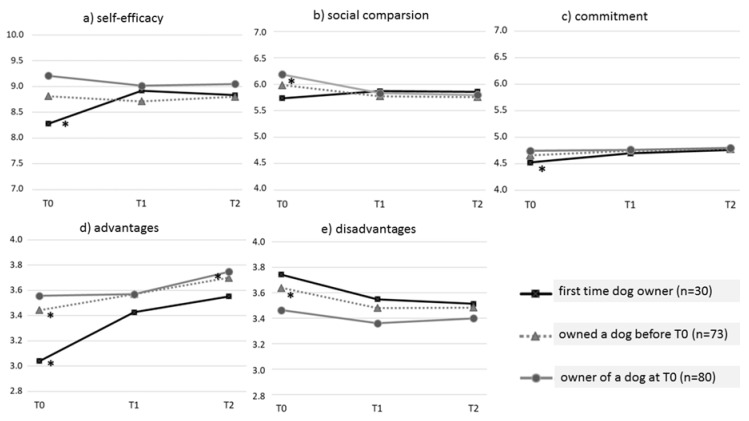
Changes over time for (**a**) self-efficacy, (**b**) social comparison, (**c**) commitment, (**d**) advantages, and (**e**) disadvantages in the three dog owning history groups. An asterix (* placed below the line) indicates a significant increase or decline between the two time points.

Figure 2a–e show the average of each of the five social cognitive variables (self-efficacy, social comparison, commitment, advantages, and disadvantages) at each of the three time points (T0, T1, and T2) for each of the three dog ownership history groups. Descriptives by group and time point can also be found in the supplemental Table S4. An asterix (*) below a group line indicates a significant increase or decrease between the two time points. GLM statistics are shown in Table 2. Significant group differences and significant decreases/increases are mentioned in the text when relevant. Full ANOVA and t-tests statistics can be found in supplemental Tables S5 and S6.

The change over time in *self-efficacy* is significantly different between the three groups as indicated by the significant interaction between self-efficacy and ownership history in Table 2. Regardless of time, the three groups differed significantly in their overall levels of self-efficacy (Table 2: main effect of ownership)This effect is most likely due to a significant difference at T0 between the groups (see Table S5). As indicated in Figure 2a, self-efficacy of unexperienced dog owners increased significantly between T0 and T1 (see Table S6). Participants who obtained an additional dog had the highest levels of self-efficacy at T0 followed by participants who owned a dog in the past, while first-time dog owners had the lowest levels of self-efficacy at T0. No group differences were present for self-efficacy scores at T1 and T2 (see Table S6).

*Social comparison* changed significantly different between the first time and experienced dog owners (see Table 2 and Figure 2b). At T0, the social comparison of current owners was significantly higher compared to that for first-time owners (see Table S5). Both experience groups declined in the early experience phase. The decline between T0 and T1 was significant for both previous and current owners, as indicated in Figure 2b and Table S6.

For changes in *commitment* over time, the pattern difference (see Figure 2c) between the groups approached significance (*p*-value = 0.071). When we combined both experience groups (previous and current) into one group, the interaction between commitment and dog ownership reached statistical significance (F(1,182) = 3.38, *p* = 0.039), indicating a significantly different pattern over time. When we compared commitment levels between the three groups, the intention to commit to the dog (T0) was significantly lower among first time owners compared to current owners (see Table S5). Commitment increased significantly between T0 and T1 among first-time owners (see Table S6).

*Perception of advantages of dog ownership* changed in a significantly different manner between the three groups (see Table 2 and Figure 2d). The increase in the early experience phase (between T0 and T1) was significant for both first time and previous owners. Previous and current owners increased significantly in their perceptions of the advantages in the extended experience phase (between T1 and T2, see Table S5). Regardless of time, a significant difference in the perception of advantages was present between the groups (indicated by the main effect of ownership; see Table 2). First time owners perceived the fewest advantages, followed by previous owners. Current owners reported the highest levels of advantages of dog ownership. However, only the differences at T0 was significant. Current owners perceived significantly more advantages of dog ownership than first time owners (see Table S4).

*For perceived disadvantages,* the pattern over time did not differ between the three dog-ownership groups (see Table 2 and Figure 2e). All three groups declined in the early experience phase and (to a lesser extent) in the extended experience phase. The main effect of ownership (Table 2) indicates an overall difference in the disadvantages between the groups, as shown in Figure 2d. First-time owners reported the most disadvantages, followed by previous owners and current owners, who reported the fewest disadvantages of dog ownership. The ANOVA results show that this effect is mainly due to a significant difference at T0 (Table S5) between first time (highest) and current dog owners (lowest). The perception of disadvantages did not differ between the three groups at T1 and T2. Contrast analyses showed a significant decline in the early experience phase (T0 to T1) among previous dog owners, but not among current or first-time dog owners (see Table S5).

**Figure 3:**
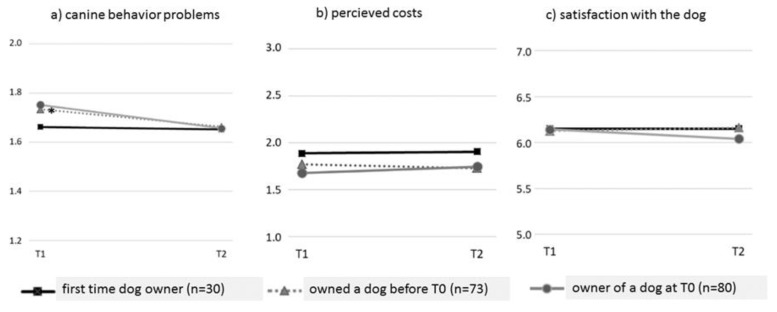
Changes over time for (**a**) canine behavioral problems, (**b**) perceived costs, (**c**) and satisfaction with the dog. An asterix (* placed below the line) indicates a significant increase or decline between two time points.

The GLM analysis indicated no difference in the patterns of *canine behavioral problem* development between the three groups (see Figure 3a and Table 3). Group analyses revealed no difference in the number of canine problems at T1 or T2 (see Table S4). For first time dog owners, the number of problematic canine behaviors stayed almost the same between T1 and T2, while for current owners, this decline was significant (see Table S5; grey solid line in Figure 3a).

The pattern for *perception of the costs of dog ownership* did not change significantly over time between the three dog ownership groups (see Table 3 and Figure 3b). No differences between the three ownership groups were observed (at T1 or at T2; see Table S4). A contrast analysis revealed no significant decrease or increase between T1 and T2 in any of the groups (see Table S5).

*Satisfaction with the dog* did not change significantly over time (see Figure 3c and Table 3), and satisfaction at T1 or T2 did not differ between the groups. Current dog owners seemed to slightly decline in their satisfaction, while previous and first time owners seemed to increase during the first six months, but these changes were not statistically significant (see Table S5).

### 3.3. Effects of Covariates

The GLM analyses revealed the effects of the following covariates: educational level, age of the dog (pup or adult), and participant age. The *self-efficacy* patterns were significantly different (F(2,181) = 3.09, *p* = 0.047) between owners with a low/intermediate (*n* = 75) and high *educational level* (*n* = 108). At all three time points, people with a high educational level had significantly greater self-efficacy compared to people with a low/intermediate education level. People with low/intermediate education started high at T0 and declined over time in their self-efficacy levels, while the opposite was observed for those with a high educational level. However, at none of the time points was the level of self-efficacy significantly different between the two education groups. Moreover, dog owners with a high educational level, regardless of time, reported significantly lower levels of perceived advantages (F(1,182) = 8.16, *p* = 0.005) than dog owners with a low/intermediate education. The group analyses revealed significant differences at all three point in time. Participants who acquired a *puppy* (*n* = 139) had significantly (F(1,182) = 12.64, *p* < 0.001) higher levels of *self-efficacy* than those who acquired an adult dog (*n* = 44) at all three time points. Those with a puppy also had significantly higher levels of *social comparison* at all three time points. In addition, participants with a puppy had significantly (F(1,182) = 4.68, *p* = 0.032) higher levels of *pet satisfaction* than participants who acquired an adult dog. This difference was only significant at T1.

*Age* had an overall effect on perception of *advantages* (F(1,182) = 5.38, *p* = 0.022) and *disadvantages* (F(1,182) = 4.32, p = 0.006). With increasing age, the participants perceived more disadvantages and fewer advantages of dog ownership. No effects of gender were present. As shown in Table 1, the first time dog owners were significantly younger compared to both previous and current owners. However, no difference was present in the percentage who acquired puppies or the difference between high versus low/intermediate educational levels between the groups. The analyses presented in this study were controlled for gender, educational level, age of the participant, and age of the dog. Detailed information about the effects of covariates can be found in the supplemental material section.

## 4. Discussion

To prevent problems, it is important have insight into how the owner–dog relationship develops over time. In the present study, we assumed that the foundations of this relationship are already present in the motivational stage before the dog is acquired. Once the dog is present, the actual experiences with the dog determine (to a large extent) the nature of the relationship in the experience phase.

Perceptions of one’s abilities and skills to take care of a dog, about one’s self and others, and one’s expectations of the advantages and disadvantages of dog ownership influence how owners perceive and experience their (prospective) relationships with dogs. Our aim was to explore how social cognitive determinants change when the dream of having a dog becomes a reality. We expected that this transition would develop differently between unexperienced and experienced dog owners since the latter group could rely on their actual experience with dogs.

Our expectations were true for all five social cognitive factors: Self-efficacy, social comparison, commitment, and perception of (dis)advantages. We showed that unexperienced owners, before acquisition of their dog, displayed lower levels of self-efficacy and social comparisons, perceived fewer advantages, expected less commitment, and perceived more disadvantages before acquisition of the dog compared to experienced dog owners. However, after six months of living with the dog, the perceptions of first time dog owners were similar to those of experienced owners. For owners who already owned a dog (and thus acquired another dog), their perceptions hardly changed.

Our results suggest that first time owners are cautious and uncertain. They may “play it safe” by not expecting too much from the dog and not underestimating the burden of dog ownership. Once they experience the relationship with their dog for six months, they have knowledge of the actual advantages, commitment, disadvantages, and their ability to handle the dog. Experienced dog owners do not seem to adjust their perceptions much; their experiences aligns with their expectations.

Among experienced dog owners, both self-efficacy and social comparisons were relatively high before acquisition of the dog. However, for experienced owners, these social comparisons declined significantly in the first six months (this was most pronounced in previous owners). This suggests that experienced owners exaggerated their skills and adjusted their estimations when they experienced life with their new dog. Possibly, experienced owners’ high social comparisons facilitated their decision to acquire another dog, despite their experience that dogs require various investments and that life with a dog “is not always about roses”.

Concerning expectations of (dis)advantages, our results agree with those of Powell et al. [8], who also showed that previous owners perceive more advantages and fewer disadvantages of dog ownership compare to unexperienced owners. However, they proposed an alternative explanation for these results: Experienced (previous/current) dog owners exhibit a bias when considering a dog because of their (unconscious) selective recall of positive experiences from previous or current ownership. Although this interpretation cannot be ruled out, our longitudinal design reveals that the high advantage and low disadvantage scores of experienced owners came true in the experience phase. Thus, experienced owners did not have to adjust their optimistic expectations, as those expectations were realistic, and unexperienced owners were cautious.

Commitment before dog acquisition was significantly lower among first time dog owners but increased significantly in the first six months of living with the dog. After eighteen months, the majority of owners showed high levels of commitment to their dog. In this study, commitment was seen as an indication of the “reward” of the dog–owner relationship. Strong commitment implies a strong motivation to invest in this relationship; it helps one endure walks in the rain and other less pleasant consequences of dog ownership. Commitment is related to emotional attachment [2], which is in turn related to ownership satisfaction [11,36]. In our sample, a moderate relationship between commitment and satisfaction with the dog was observed (T1: r = 0.37, T2: r = 0.42). As recently shown in [37], the beneficial effects of dog ownership are mediated by the positive effects of shared activities, such as walking or playing. Herwijnen et al. [36] found no evidence that shared activities increased relationship satisfaction. They did find, however, that a high perception of costs (MDORS subscale) was associated with aggression and/or disobedience by the dog and hence with decreased satisfaction with the dog. More research is needed to understand which factors are involved in ownership satisfaction, since this factor may provide strategies to improve the relationship between humans and dogs.

Mondelli et al. [38] showed that experienced dog owners were more likely to return a dog to an animal shelter because of behavioral problems, which suggests that experienced owners are less tolerant of misbehavior. Their experience may also make such owners more realistic, understanding, for example, that certain behavioral problems cannot be solved by themselves. We found a significant decline in canine behavioral problems among current dog owners, which might be explained by their ability to adequately adjust problematic behavior or by corrections from the other dogs present in the household [39]. Earlier cross-sectional research showed that first time dog owners report a higher prevalence of problematic behaviors, such as fear, over-excitability, and owner-directed aggression [40,41,42]. We found no differences in the number of reported behavioral problems between experienced and unexperienced owners, and a post-hoc analysis of separate behavioral problems did not support the aforementioned findings. Differences between previous studies and ours might be due to the nature of the samples. A recent study by Dinwoodie et al. [43] examined canine behavioral problems in an international sample of over 4000 owners and showed that the median number of reported behavioral problems per dog was 2 (ranging from 0 to 12). In our sample, the median was 1.7 for T1 and 1.6 for T2 (range 0 to 14), which suggests a lower prevalence of behavioral problems in our sample.

Changes in social cognitive determinants were assessed at three points in time, and our findings show that most changes occurred in the first six months of dog ownership. Since the perceived costs and satisfaction did not change between six and eighteen months of dog ownership, the perception of the costs of and satisfaction with the dog were also presumably stable after six months. The present cohort was comprised of dog-owners who kept their dogs, which might explain why limited evaluations of this relationship were observed.

In sum, the results of the present study show that changes in beliefs mainly occur in the first months of dog ownership (in the early experience phase) and do not change notably in the following year (the extended experience phase). Moreover, concerning perceived costs, satisfaction with the dog, and canine behavioral problems, hardly any changes occurred during the extended experience phase. Our results suggest that after six months of living with a dog, a more or less stable relationship is formed.

### Limitations of the Study

Our study is subject to several limitations. Our sample was not entirely representative of the population of dog owners in the Netherlands [44]. First, while most dogs in the Netherlands are owned by families with children, only one third of our respondents were part of a family with children. Second, although most dogs are mongrels, almost three quarters of our respondents owned purebred dogs. Moreover, highly educated women were over-represented in our sample. This is common in animal-related research, partly due to the general tendency of women to have a more positive attitude towards animals than men [45]. In addition, people with low or intermediate levels of education were under-represented in our sample. Fourth, as is common in studies on human–animal relationships, this research unintentionally focused on highly engaged pet owners, which may have biased our results. Fifth, it might be possible that some participants dropped out because they were disappointed with the dog or their relationship with it and thus discarded the dog. We have no detailed knowledge about this possibility.

Not all measures had good internal consistency. The alpha of the satisfaction scale was especially questionable [27] at T1 (α = 0.46). The internal consistency of this scale at T2 was 0.73. The post hoc and correlation between both measurements was moderate (r = 0.46). Although it is possible that the uneven internal consistency of the satisfaction scale reflected the owners’ different interpretations of the items at T1 and T2 (which would explain the different alpha results), our findings must be interpreted with care.

Notably, our theoretical model does not exactly reflect our data assessment. Our theoretical model consists of two stages: The motivational phase (in which the decision to acquire a dog unfolds) and the experience phase (where people actually experience their new dog). The motivation phase ends exactly at the moment the dog is acquired. However, we did not collect our data immediately at this moment. Although we did this on purpose (since the first months of dog ownership are still a period of instability, where beliefs and attitudes might change back and forth), this delay could be considered a limitation of our study design.

A last aspect that needs to be considered when interpreting the present findings is that the sample recruitment method may have influenced the results. Our aim to hear from “people planning to acquire a dog within one year” may have led to the exclusion of people who would have scored negatively in advantages, social norms, and self-efficacy regarding dogs, as well as those with little preparation activity. This may have led to less variance and, consequently, less covariance between variables.

## 5. Conclusions

People with current or previous dog ownership can rely on their experience when deciding to get a new dog, but this knowledge is unavailable to first time dog owners. Our longitudinal design showed that the social cognitive factors involved in the establishment of a relationship with a dog change differently over time depending on dog ownership history. Secondly, we showed that these changes mainly occur in the transition from the motivational (where the relationship with the dog is still an illusion) to the experience phase (when the relationship with the dog has become a reality) but that these changes remain steady when the relationship endures. Our findings illustrate the importance of considering dog ownership history in studies on human–animal relationships. Longitudinal studies are important because they increase our knowledge about the underlying social cognitive processes that drive human behavior and shape our relationships with animals. A better understanding of the underlying (unconscious) motivations and perceptions that drive human behavior and how they change over time may provide starting points for interventions to prevent problems and improve the welfare of both animals and humans.

## Figures and Tables

**Table 1:** Group differences according to experience with dogs.

Ownership	First	Previous	Current	Chi-Square Test/Analysis of Variance			
*n*	*n* = 30	*n* = 73	*n* = 80	F or Pearson X^2^	df	*p*-value	difference
% female	90%	85%	89%	X^2^ = 0.72	2183	0.697	
% high education	77%	53%	58%	X^2^ = 4.88	2183	0.087	
% puppy	77%	72%	79%	X^2^ = 0.80	2183	0.670	
age (years)	36.8 (10.5)	46.7 (13.5)	42.2 (11.8)	F = 7.15	1182	0.001	first-timers, younger

First: first time dog owner (*n* = 30); previous: owned a dog before T0 (*n* = 73); current: owner of a dog at T0 (*n* = 80).

**Table 2:** General Linear model of the changes in social cognitive factors over time (T0, T1, and T2).

Factor	Interaction Effect Factor × Ownership			Main Effect Ownership			Main Effect Time		
Test statistic	F	df	*p*-value	F	df	*p*-value	F	df	*p*-value
Self-efficacy	4.99	4179	0.001	4.21	2181	0.016	0.50	2181	0.603
Social comp	3.79	4179	0.005	0.66	2181	0.520	3.72	2181	0.026
Advantages	4.57	4179	0.002	4.58	2181	0.012	0.80	2181	0.436
Disadvantages	0.62	4179	0.651	3.23	2181	0.042	0.81	2181	0.444
Commitment	2.22	4179	0.071	1.76	2181	0.175	0.31	2181	0.708

The abbreviation “Social comp.” refers to “Social comparison score”.

**Table 3:** General Linear modeling of changes in the outcome measures over time (T1 and T2).

Factor	Factor*Ownership			Main Ownership			Main Time		
	F	df	*p*-value	F	df	*p*-value	F	df	*p*-value
Behavioral problems	0.80	1,182	0.452	0.193	2,181	0.825	0.059	2,181	0.809
Perceived costs	1.40	1,182	0.249	0.93	2,181	0.398	2.27	2,181	0.134
Satisfaction	0.58	1,182	0.562	0.22	2,181	0.802	0.41	2,181	0.525

## Supplementary Materials

The following are available online at https://www.mdpi.com/2076-2615/10/5/772/s1, Table S1: Overview of canine behavioral problems; Table S2: Differences between ownership groups for all variables by time point; Table S3: Rise and fall between time points for all variables by dog ownership group; Table S4. Descriptive elements by group and time point; Table S5. Rise and fall between time points for all variables by dog ownership group; Table S6. Differences between the ownership groups for all variables by time point.
