# Supplementary material for: Expectations Versus Reality: Long-Term Research on the Dog–Owner Relationship
Source: Animals (Basel). 2020 Apr 29;10(5):772. doi: 10.3390/ani10050772 (PMC7278369; doi:10.3390/ani10050772)
Supplement: Supplementary file 1 [file animals-10-00772-s001.pdf]

animals

Supplementary Materials: Expectations versus Reality: Long-Term Research on the Dog–Owner Relationship

Esther M.C. Bouma *, Lonneke M. Vink † and Arie Dijkstra

Department of Social Psychology, Faculty of Behavioural and Social Sciences, University of Groningen, 9712 CP Groningen, the Netherlands; arie.dijkstra@rug.nl

* Correspondence: e.m.c.bouma@rug.nl

† Deceased.

Table S1. Advantages of dogs and dog ownership.

What Advantages Do You see in Dogs/Dog Ownership?
1. Having company
2. Feeling more confident about myself
3. Feeling more safe
4. Having a companion
5. Feeling more connected with nature
6. Feeling less lonely
7. Being outside more often to clear my head
8. Getting more exercise
9. Having less stress
10. Being more physically fit
11. Getting sick less often
12. Having someone to talk to
13. Being taken more seriously by other people
14. Having more structure in my life
15. Have someone to play with
16. Having more social contact with other people
17. Have a better mood
18. Experiencing more livelihood around the house
19. Feeling less depressed
20. Have someone who is there for me unconditionally
21. Feeling more complete
22. Having my house guarded
23. Having fewer physical complaints
24. Feeling more happy
25. Feeling more valued by other people

Table S2. Disadvantages of dogs and dog ownership.

1.	Because of my dog… I have less freedom to go away spontaneously
2.	I have to get out of the house multiple times a day, despite the weather
3.	I have an extra and significant responsibility in my life
4.	I have to spend much time on my dog's care
5.	I have to spend more money
6.	I have to clean my house more often
7.	I have to get up early to walk my dog
8.	I have to clean dog feces
9.	I have to plan my life more
**10.**	I have a house that smells less nice

Table S3. Overview of canine behavioral problems.

1. Not responding to stop commands
2. Bad eating manners
3. Not listening when called upon
4. Pulling the leash
5. Inappropriate soiling
6. Inappropriate soft/playful biting
7. General disobedience
8. Aggression toward other dogs
9. Aggression toward unfamiliar people
10. Aggression toward familiar people
11. Inappropriate digging
12. Destructive behavior
13. Inappropriate chewing or biting inedible objects
14. Hyperactivity
15. Inappropriate barking
16. Jumping onto people

Table S4. Descriptive elements by group and time point.

Group		Overall (*n*=183)		First (*n* = 30)		Previous (*n* = 73)		Current (*n* = 80)	
**Variable**	**Range**	**Mean**	**SD**	**Mean**	**SD**	**Mean**	**SD**	**Mean**	**SD**
Self-efficacy T0	1–10	8.9	10.03	8.27	13.05	8.82	0.95	9.26	0.79
Self-efficacy T1	1–10	8.9	0.93	8.98	0.90	8.73	10.04	9.12	0.85
Self-efficacy T2	1–10	8.92	0.97	8.83	0.93	8.80	10.57	9.05	0.88
Advantages T0	1–25	3.43	0.55	3.04	0.55	3.44	0.59	3.56	0.54
Advantages T1	1–25	3.55	0.51	3.43	0.54	3.57	0.47	3.57	0.53
Advantages T2	1–25	3.70	0.51	3.55	0.41	3.70	0.53	3.75	0.47
Disadvantages T0	1–10	3.58	0.5	3.74	0.59	3.64	0.49	3.46	0.51
Disadvantages T1	1–10	3.44	0.58	3.55	0.48	3.48	0.51	3.36	0.63
Disadvantages T2	1–10	3.45	0.53	3.51	0.72	3.48	0.51	3.40	0.57
Social comparison T0	1–7	6.03	0.72	5.74	0.72	5.99	0.68	6.19	0.71
Social comparison T1	1–7	5.82	0.69	5.87	0.86	5.78	0.68	5.83	0.70
Social comparison T2	1–7	5.79	0.72	5.86	0.33	5.76	0.69	5.80	0.70
Commitment T0	1–5	4.67	0.32	4.52	0.32	4.66	0.34	4.74	0.29
Commitment T1	1–5	4.75	0.29	4.70	0.30	4.75	0.27	4.76	0.29
Commitment T2	1–5	4.79	0.32	4.77	0.26	4.78	0.28	4.80	0.36
Problematic behaviors T1	1–16	1.73	0.34	1.66	0.23	1.73	0.35	1.72	0.37
Problematic behaviors T2	1–16	1.66	0.30	1.65	0.26	1.66	0.27	1.68	0.34
Satisfaction T1	1–7	6.11	0.98	1.89	0.53	1.77	0.57	1.68	0.54
Satisfaction T2	1–7	6.14	0.80	1.91	0.50	1.73	0.57	1.75	0.54
Perceived costs T1	1–5	1.75	0.55	6.15	0.75	6.16	0.98	6.04	1.06
Perceived costs T2	1–5	1.77	0.56	6.15	0.66	6.13	0.83	6.14	0.85

Table S5. Differences between the ownership groups for all variables by time point.

Variable	F-value	df	*p*-value	Sig. Difference
Self-efficacy T0	11.08	2,183	$p < 0.001$	first < previous < current
Self-efficacy T1	2.07	2,183	0.129	
Self-efficacy T2	1.39	2,183	0.250	
Advantages T0	10.47	2,183	$p < 0.001$	first < previous < current
Advantages T1	1.01	2,183	0.367	
Advantages T2	1.60	2,183	0.205	
Disadvantages T0	4.47	2,183	0.013	first > current
Disadvantages T1	1.47	2,183	0.234	
Disadvantages T2	0.71	2,183	0.496	
Social comparison T0	4.71	2,183	0.010	first < current
Social comparison T1	0.24	2,183	0.782	
Social comparison T2	0.20	2,183	0.821	
Commitment T0	5.51	2,183	0.005	first < current
Commitment T1	0.60	2,183	0.550	
Commitment T2	0.15	2,183	0.859	
Problematic behavior T1	0.74	2,183	0.480	
Problematic behavior T2	0.02	2,183	0.985	
Satisfaction T1	0.12	2,183	0.988	
Satisfaction T2	0.32	2,183	0.726	
Perceived costs T1	1.68	2,183	0.190	
Perceived costs T2	1.14	2,183	0.324	

Table S6. Rise and fall between time points for all variables by dog ownership group.

Variable	First (*n* = 30)			Previous (*n* = 73)			Current (*n* = 80)		
	t-value	df	*p*-value	t-value	df	*p*-value	t-value	df	*p*-value
**T0 to T1**									
Self-efficacy	-3.25	1,29	0.003	0.77	1,72	0.442	1.81	1,79	0.075
Advantages	-3.55	1,29	0.001	-2.77	1,72	0.007	-0.23	1,79	0.821
Disadvantages	1.71	1,29	0.098	2.77	1,72	0.007	1.81	1,79	0.075
Social comparison	-1.08	1,29	0.289	2.47	1,72	0.016	4.65	1,79	< 0.001
Commitment	-2.06	1,29	0.048	-2.19	1,72	0.032	-0.74	1,79	0.464
**T1 to T2**									
Self-efficacy	1.01	1,29	0.320	-0.64	1,72	0.520	-0.33	1,79	0.740
Advantages	-1.90	1,29	0.067	-3.27	1,72	0.002	-4.32	1,79	< 0.001
Disadvantages	-0.38	1,29	0.710	-0.04	1,72	0.967	-0.69	1,79	0.494
Social comparison	0.10	1,29	0.925	0.16	1,72	0.871	0.54	1,79	0.592
Commitment	-1.70	1,29	0.100	0.10	1,72	0.290	-1.25	1,79	0.217
Problematic behavior	0.23	1,29	0.818	1.96	1,72	0.054	2.98	1,79	0.004
Perceived costs	-0.23	1,29	0.822	0.79	1,72	0.430	-1.13	1,79	0.264
Satisfaction	0.00	1,29	1.000	-0.34	1,72	0.737	0.86	1,79	0.390

Detailed Information on Significant Effects of Covariates

GLM analyses revealed effects of the following covariates: educational level, age of the dog (pup or adult) and of participants' age. No effects of gender were present.

With regard to **educational level**, *self-efficacy* patterns were significantly ($F(2,181)=3.09$, $p=0.047$) different between owners with a low/intermediate (n=75) and owners with a high educational level (n=108). At all three time points, people with a high educational level had significantly more self-efficacy (M(SD) T0= 8.79 (10.57), T1 = 8.9.4 (0.93), T2 = 8.97 (0.96)) compared to people with a low/intermediate education level (M(SD) T0= 9.05 (0.90), T1 = 8.83 (0.94), T2 = 8.83 (0.97). People with a low/intermediate education start high at T0 and decline over time in their self-efficacy levels while this is the other way around for people with a high educational level. However, at none of the time points the level of self-efficacy is significantly different between the two education groups (T0: F(1,182 = 3.16, p =

0.077; T1: F(1,182 = 0.58, p = 0.448; T2: F(1,182 = 0.914, p = 0.340). Moreover, dog owners with a high education (n=108: M(SD) T0 = 3.32 (0.56), T1 = 3.46 (0.49), T2 = 3.63 (0.53), report regardless of time, significantly lower levels of *perceived advantages* (F(1,182) = 8.16, p = 0.005) than dog owners with a (low/intermediate education (n = 75: M(SD) T0 = 3.59 (0.50), T1 = 3.66 (0.50), T2 = 3.79 (0.47). Group analyses revealed significant differences at all three point in time for perceived advantages (T0: F(1,182 = 11.27, p = 0.001; T1: F(1,182 = 7.11, p = 0.008; T2: F(1,182 = 4.30, p = 0.040).

Participants who acquired a **puppy** (n=139) have overall significantly (F(1,182) = 12.64, p < 0.001) higher levels of *self-efficacy* (M(SD) T0= 8.99 (0.99), T1 = 90.36 (0.85), T2 = 90.40 (0.90) compared to people who acquired an adult dog (n = 44, M(SD) T0= 8.61 (0.99), T1 = 8.45 (10.39), T2 = 85.23 (10.67). At all three time points levels are significantly higher (T0: F(1,182 = 4.69, p = 0.032; T1: F(1,182 = 13.99, p < 0.001; T2: F(1,182 = 10.34, p = 0.002). Participants who acquired a puppy (n=139) also had overall significantly (F(1,182) = 9.88, p = 0.002) higher levels of *social comparison* (M(SD) T0 = 6.12 (0.67), T1 = 5.91 (0.68), T2 = 58.8 (0.65) compared to people who acquired an adult dog (n=44, M(SD) T0 = 5.81 (0.84), T1 = 5.54 (0.67), T2 = 5.52 (0.87). At all three time points levels are significantly higher (T0: F(1,182 = 5.93, p = 0.016; T1: F(1,182 = 9.87, p = 0.002; T2: F(1,182 = 8.75, p = 0.004). And finally, participants who acquired a puppy (n=139) had overall significantly (F(1,182) = 4.68, p = 0.032) higher levels of *pet satisfaction* (M(SD) T1 = 6.21 (0.73), T2 = 6.17 (0.93)) compared to people who acquired an adult dog (n=44, M(SD) T1 = 5.93 (10.05), T2 = 5.90 (10.99). This difference was only significant at T1 (F(1,182 = 4.05, p = 0.046) but not at T2 (F(1,182 = 2.66, p = 0.105).

Age had an overall effect on perception of *advantages* (F(1,182) = 5.38, p = 0.022) and perception of *disadvantages* (F(1,182) = 4.32, p = 0.006). With increasing age, participants perceive more disadvantages and less advantages of dog ownership. When we compare four age groups (18-30, 31-45, 46-60 and 60 years or older) no significant difference between the groups were present, not for advantages (T0: F(3,180) = 1.64, p = 0.182, T1: F((3,180)= 2.13, p = 0.10, T2: F((3,180)= 1.30, p = 0.78) and neither for advantages (T0: F(3,180) = 1.60, p = 0.192, T1: F((3,180)= 1.37, p = 0.255, T2: F((3,180)= 1.67, p = 0.175).
